# Supplementary material for: HDAC1 and HDAC2 integrate checkpoint kinase phosphorylation and cell fate through the phosphatase-2A subunit PR130
Source: Nat Commun. 2018 Feb 22;9:764. doi: 10.1038/s41467-018-03096-0 (PMC5823910; doi:10.1038/s41467-018-03096-0)
Supplement: Supplementary file 1 — Supplementary Information [file 41467_2018_3096_MOESM1_ESM.docx]

|  | **HU** | **MS** | **MS+HU** |
| --- | --- | --- | --- |
| *PPP2R3A* | 0,98 | 1,79 | 1,96 |
| *PPP2CB* | 1,16 | 1,06 | 1,19 |
| *PPP2R2B* | 1,12 | 0,80 | 1,15 |
| *PPP2R5D* | 0,97 | 0,98 | 1,10 |
| *PPP2R1B* | 1,10 | 1,07 | 1,09 |
| *PPP2CA* | 0,94 | 0,99 | 1,05 |
| *PPP2R3C* | 0,87 | 0,71 | 1,04 |
| *PPP2R2A* | 1,13 | 1,12 | 1,04 |
| *PPP2R2D* | 1,09 | 0,75 | 1,03 |
| *PPP2R2C* | 0,95 | 0,98 | 1,03 |
| *PPP2R5C* | 0,79 | 1,05 | 1,01 |
| *PPP2R5A* | 0,89 | 1,03 | 0,89 |
| *PPP2R5C* | 0,89 | 0,86 | 0,86 |
| *PPP2R3B* | 1,06 | 0,97 | 0,85 |
| *PPP2R5E* | 0,82 | 0,83 | 0,78 |
| *PPP2R1A* | 0,96 | 0,93 | 0,72 |
| *PPP2R5B* | 1,27 | 1,59 | 1,79 |
| *PPP2R2B* | 1,12 | 0,80 | 1,15 |
| *PPP2R4* | 0,83 | 0,62 | 0,51 |
| *RRM2* | 1,54 | 0,85 | 1,74 |
| *RRM2B/p53R2* | 1,60 | 1,15 | 1,53 |
| *RAD50* | 0,78 | 0,92 | 0,85 |
| *RAD51* | 1,23 | 0,51 | 0,96 |
| *TP53BP1* | 0,84 | 0,88 | 0,83 |
| *CHEK1* | 1,02 | 0,60 | 0,83 |
| *ATM* | 1,11 | 0,84 | 0,94 |

Expression rel. to ctrl


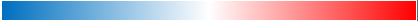


2

0

1

**Supplementary Table 1**: **mRNA expression of selected genes acquired by microarray analysis**. HCT116 cells were treated with 1 mM hydroxyurea (HU) and 2 µM MS-275 (MS) for 24 h. Values are presented as expression levels relative to untreated HCT116 cells (Ctrl).


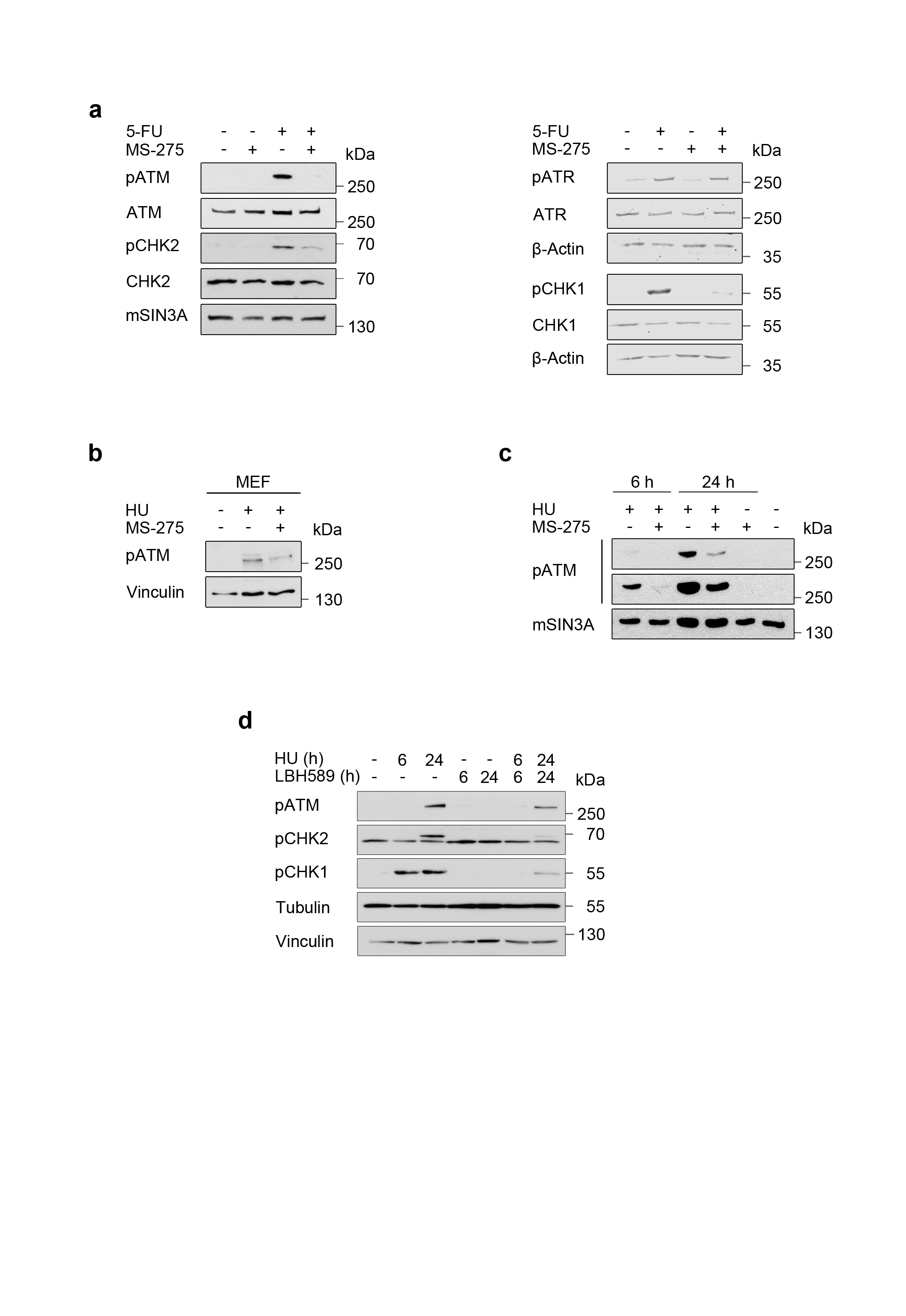


**Supplementary Figure 1. HDACi impair checkpoint kinase signalling in response to various replicative stress inducers and in different cellular systems.**

(**a**) 5-Fluorouracil (5-FU; 5 µM) and MS-275 (2 µM) were added alone or in combination for 24 h to HCT116 cells. Individual proteins and their phosphorylated species were detected by Western blot with mSIN3A and β-Actin serving as loading control (n=3). (**b**) MEF cells were incubated with hydroxyurea (HU) (1 mM) and/or MS-275 (2 μM) for 24 h. Phosphorylation of ATM was determined by Western blot analysis. Vinculin was used as loading control (n=3). (**c**) RKO cells were incubated with HU (1 mM) and MS-275 (2 µM) for 6 and 24 h. Single MS-275 treatment was carried out for 24 h. ATM phosphorylation as well as mSIN3A (loading control) expression was assessed by immunoblot (n=2). (**d**) Treatment with HU (1 mM) and/or LBH589 (100 nM) was carried out for 6-24 h in HCT116. Phosphorylated forms of ATM, CHK1, and CHK2 were detected by Western blot. Tubulin and Vinculin served as loading control (n=2).


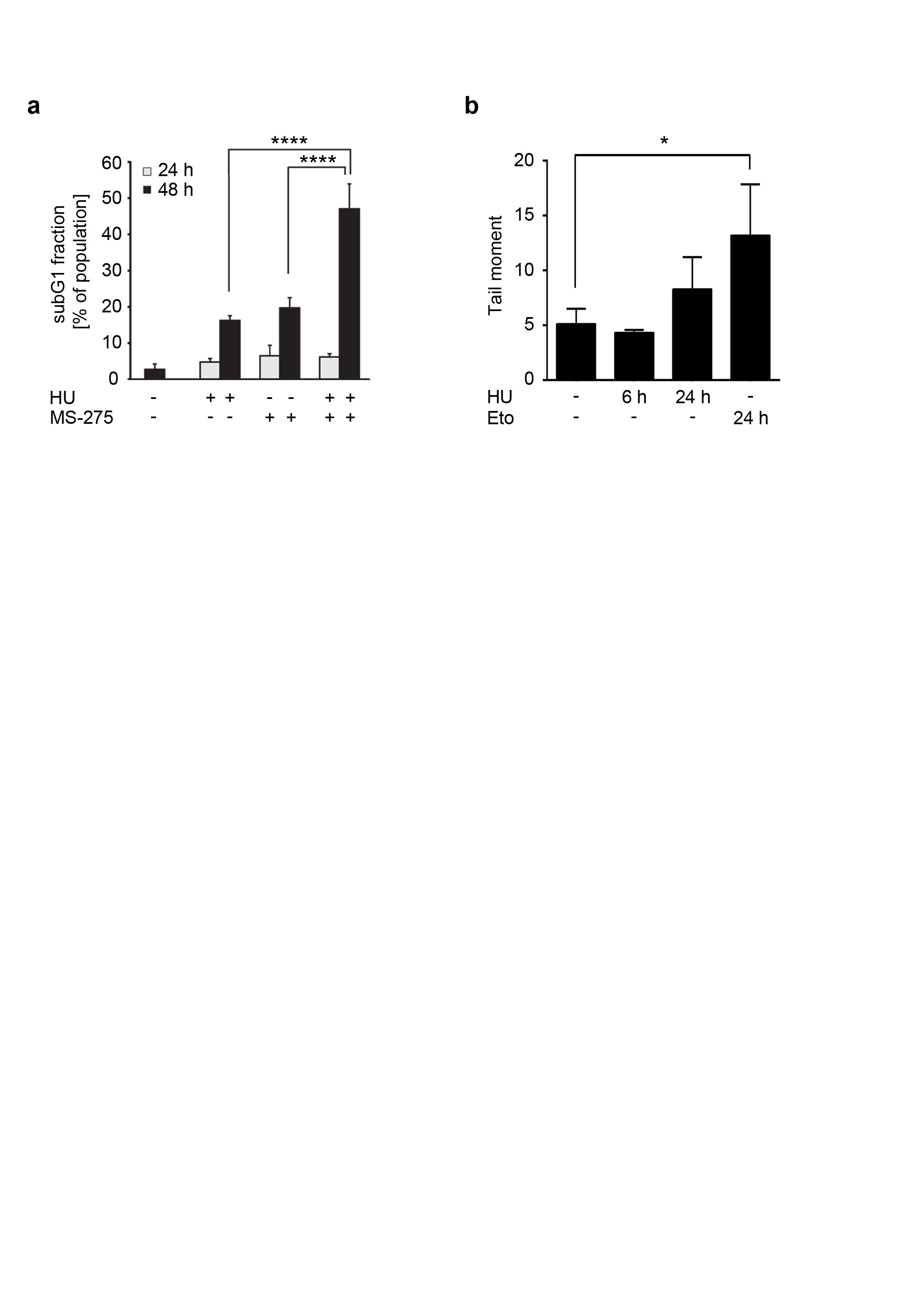


**Supplementary Figure 2. HDACi promote cell death upon HU treatment.**

(**a**) Fractions of HCT116 cells in subG1 were determined by flow cytometry after PI staining. Cells were left untreated as control or were incubated with 1 mM hydroxyurea (HU), 2 μM MS-275 or both for 24 and 48 h. Results show mean±SD of three independent experiments (One-way ANOVA, **** *P*<0.0001). (**b**) Neutral comet assay was performed with HCT116 cells treated with 1 mM HU for 6-24 h or 10 µM etoposide (Eto) for 24 h. At least 50 cells were counted per condition. Results show mean±SD of three independent experiments (One-way ANOVA, * *P*<0.05).


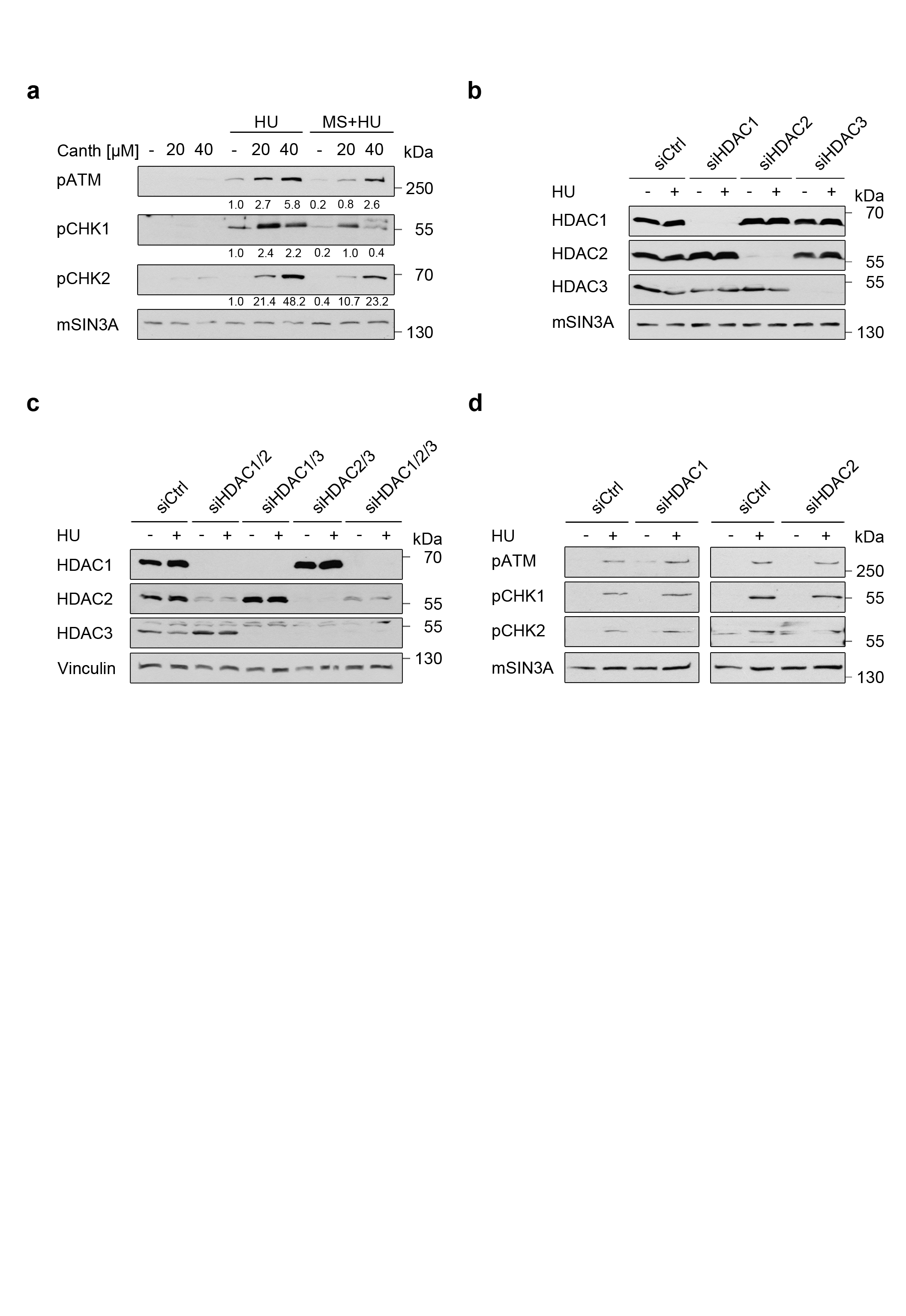


**Supplementary Figure 3. HDAC1 and HDAC2 repress the PP2A-B subunit PR130 to regulate checkpoint kinase phosphorylation.**

(**a**) HCT116 cells were left untreated a control or were incubated with 2 μM MS-275 and/or 1 mM hydroxyurea (HU) for 24 h. Cantharidin (Canth; 20-40 µM) was added for additional 4 h. Proteins were detected with specific antibodies by Western blotting. mSIN3A served as loading control (n=3). (**b**) Single knockdown of HDAC1, 2 and 3 was performed by transient transfection with respective siRNAs for 48 h. Subsequently, medium was changed and cells were left untreated as control or 1 mM HU was added for further 24 h. Knockdown efficiency was assessed by Western blot (n=3). (**c**) Efficiencies of double and triple knockdowns with siRNAs against HDAC1, 2 and/or 3. Time schedule of transfection and treatment as described in (**b**) (n=3). (**d**) Western blots show the effect of single knockdown of HDAC1 and 2, respectively, on checkpoint kinase phosphorylation. Same protocol was used as in (**b**). Obtained whole cell lysates were used for protein detection. mSIN3A was used as loading control (n=3).


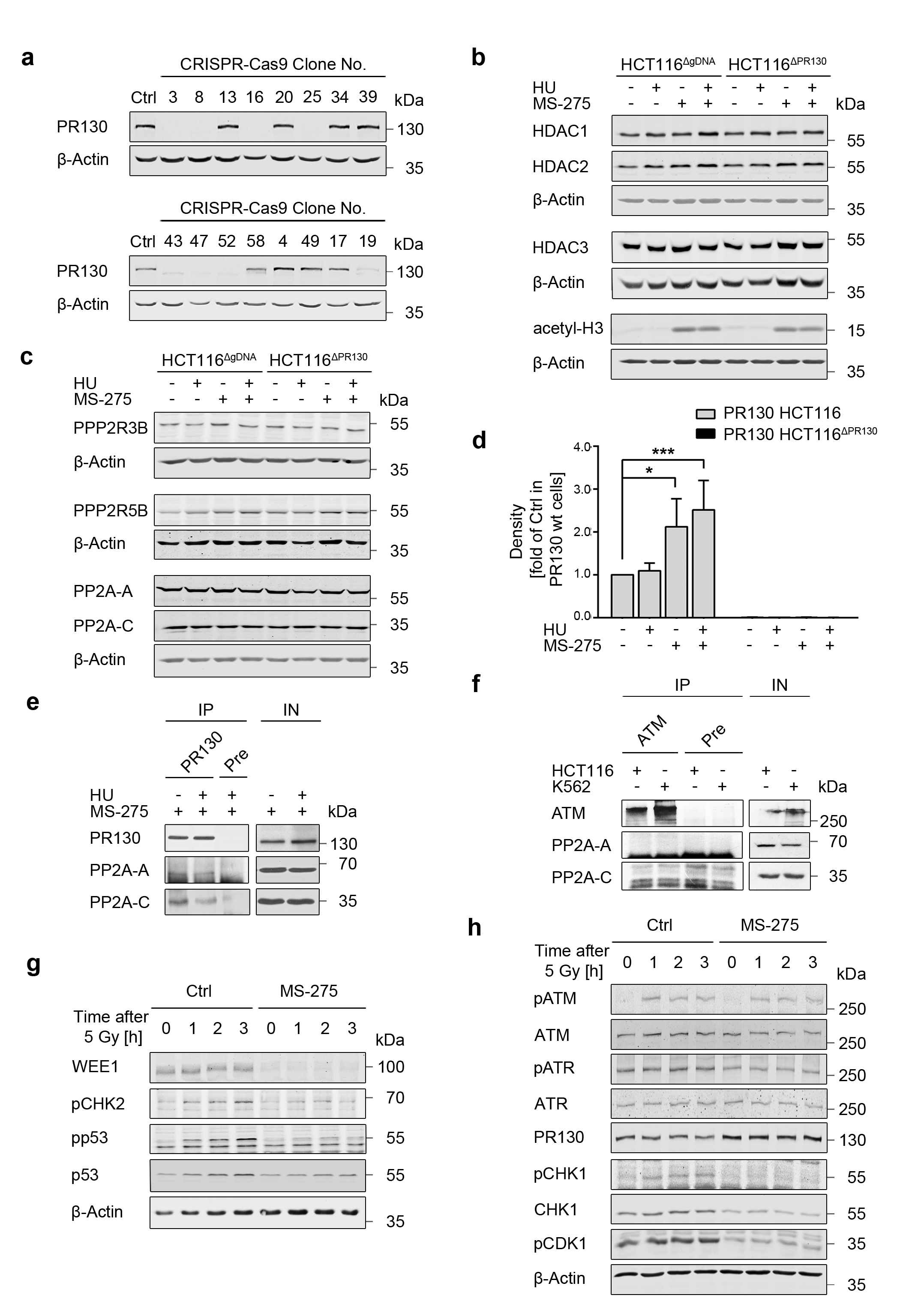


**Supplementary Figure 4. PR130-knockout using the CRISPR-Cas9 technology.**

(**a**) Western blot analysis of PR130 expression in different HCT116^ΔPR130^ clones. HCT116^ΔgRNA^ cells were used as control for PR130 expression. β-Actin served as loading control (n=2). (**b**) HCT116^ΔgRNA^ and HCT116^ΔPR130^ cells were treated with 1 mM hydroxyurea (HU) and 2 µM MS-275 for 24 h. Western blot analyses of whole cell lysates were performed to detect HDAC1, HDAC2, HDAC3, β-Actin (loading control), and acetylated histone 3 (acetyl-H3) (n=3). (**c**) Cells were treated as stated in (**b**) and Western blot was done as indicated (n=3). (**d**) Quantitative analysis of PR130 levels detected by Western blot analysis in HCT116^ΔPR130^ and HCT116 control cells. Treatment was performed as described in (**b**). Data were normalised to respective loading controls and displayed as relative to untreated cells. Results are presented as mean±SD (n=5; One-way ANOVA; * *P*<0.05, *** *P*<0.001). (**e**) HCT116 cells were treated and processed as stated in the legend to Fig. 6d. Immunoprecipitates (IP) of PR130 were tested for PR130 and the A and C subunits of PP2A (n=2) (Pre, pre-immune IgG; IN, input). (**f**) ATM was precipitated from HCT116 and K562 cells, as described in the Methods section. Immunoblot was performed for ATM, PP2A-A, and PP2A-C. (**g**) Cells were either pre-incubated with 2 µM MS-275 of left untreated for 24 h and subsequently exposed to 5 Gy. Individual plates were harvested after 0, 1, 2, 3 h. Indicated proteins were detected via Western blot (n=3). (**h**) Cells treated as described in (**f**). Indicated proteins were detected via Western blot. β-Actin served as loading control (n=3).


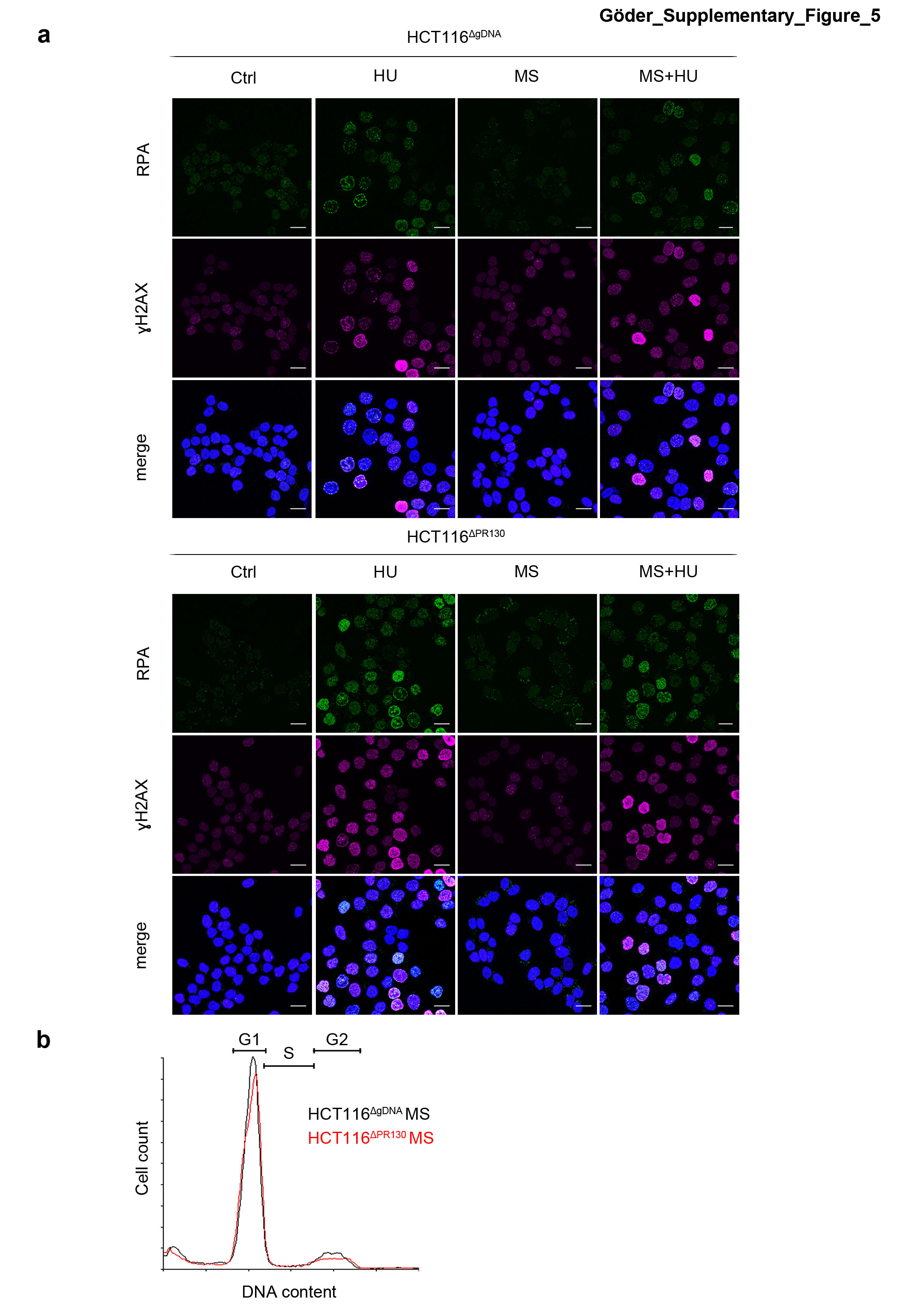


**Supplementary Figure 5. PR130 influences cell fate and DNA damage response.**

(**a**) HCT116^ΔgRNA^ (upper panel) and HCT116^ΔPR130^ (clone #16; lower panel) cells were incubated with 1 mM HU and 2 µM MS-275 for 24 h. Cells were fixed and incubated with RPA- (green) and ɣH2AX-specific antibodies (magenta). Co-staining with secondary antibody was performed using Alexa Fluor-488- (RPA) and Cy3- (ɣH2AX) coupled antibodies and TO-PRO3 was used to visualize nuclei. Images represent uncropped versions of micrographs shown in figure 8a (n=3; scale bar, 20 µm). (**b**) Flow cytometry analyses of HCT116^ΔgRNA^ (black) and HCT116^ΔPR130^ (clone #3; red) treated with 2 µM MS-275 for 40 h. DNA content was stained using PI. Representative histogram is shown (n=3).


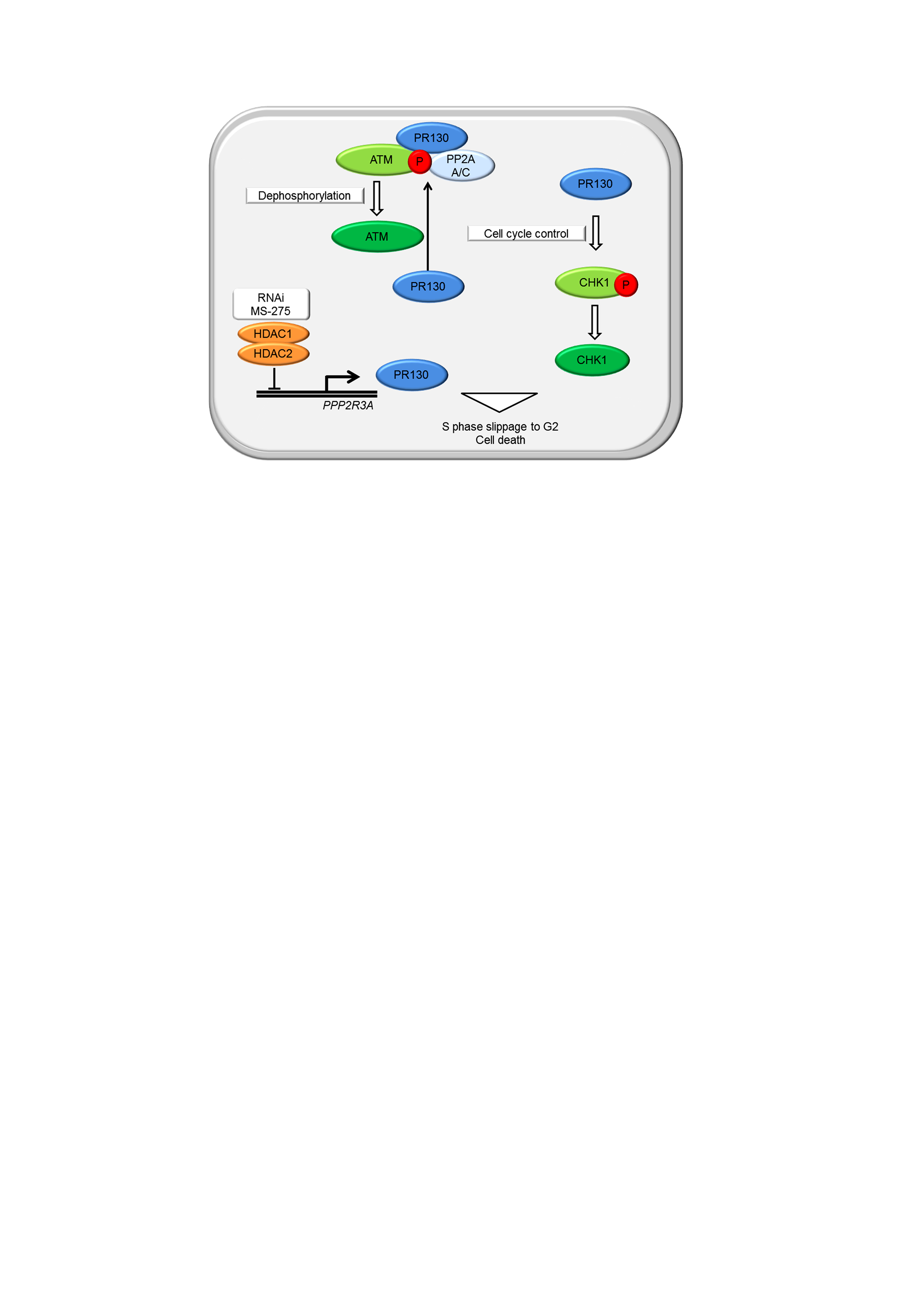


**Supplementary Figure 6. Inhibition of HDAC1 and HDAC2 modulates the DNA damage response**.

Simultaneous inhibition by MS-275 or genetic elimination of HDAC1 and HDAC2 induces expression and acetylation of the PP2A regulatory subunit PR130. The increase in PR130 results in a dephosphorylation of ATM by the PP2A holoenzyme. The control of cell cycle progression by PR130 affects the traversal of cells from G1 to S phase and PR130 reduces the phosphorylation of CHK1. Upon diminished kinase activity of ATM and CHK1, S phase arrest can no longer be maintained and cells progress toward G2 phase and mitosis subsequently undergoing caspase-dependent cell death.


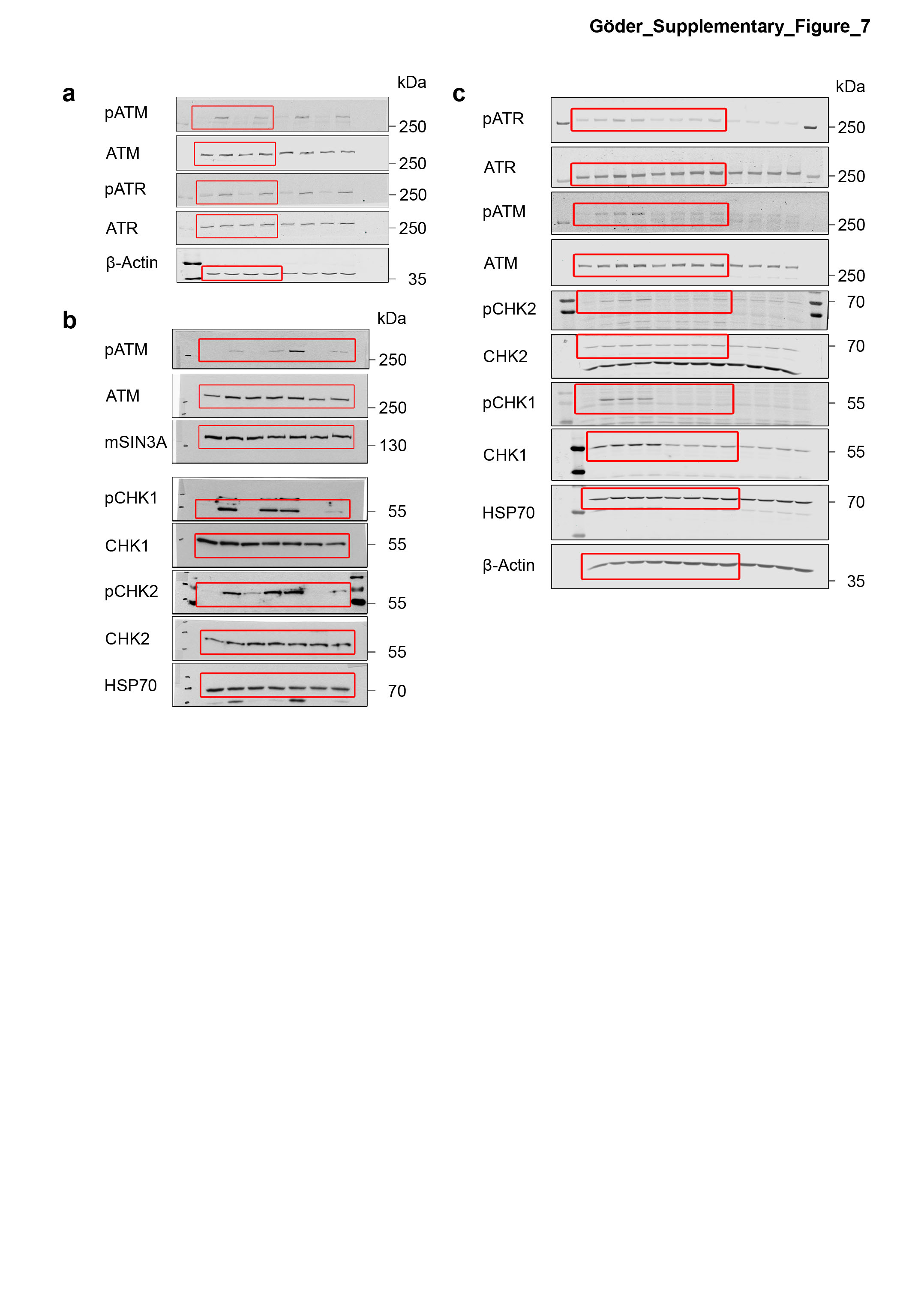


**Supplementary Figure 7. Uncropped Western blot images for data shown in Figures (a) 1a, (b) 1c, and (c) 1e.** Red boxes mark the parts of the Western blot images that are shown in the indicated figures.


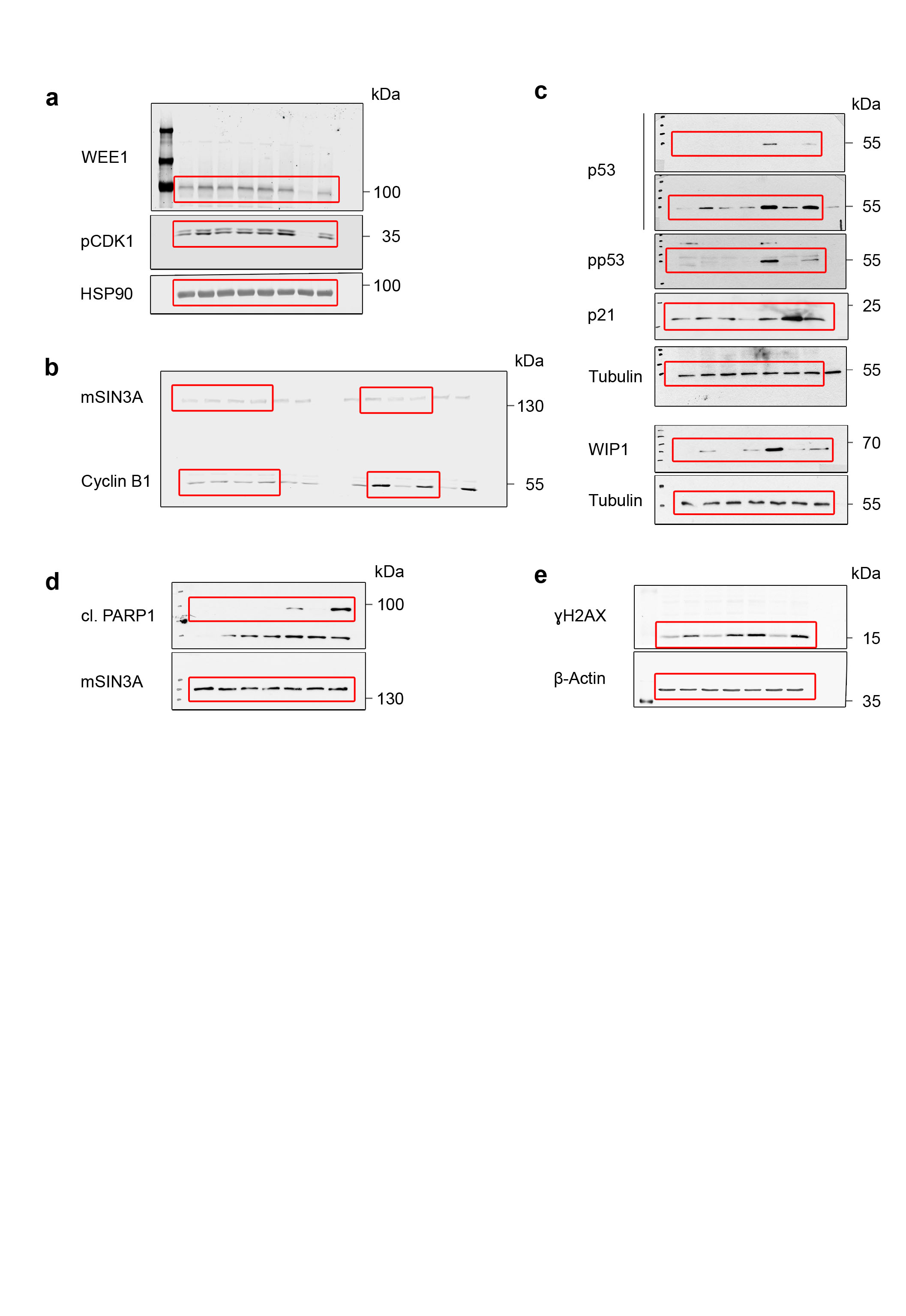


**Supplementary Figure 8. Uncropped Western blot images for data shown in Figures (a) 2f, (b) 2g, (c) 2h, (d) 3b, and (e) 3f.** Red boxes mark the parts of the Western blot images that are shown in the indicated figures.


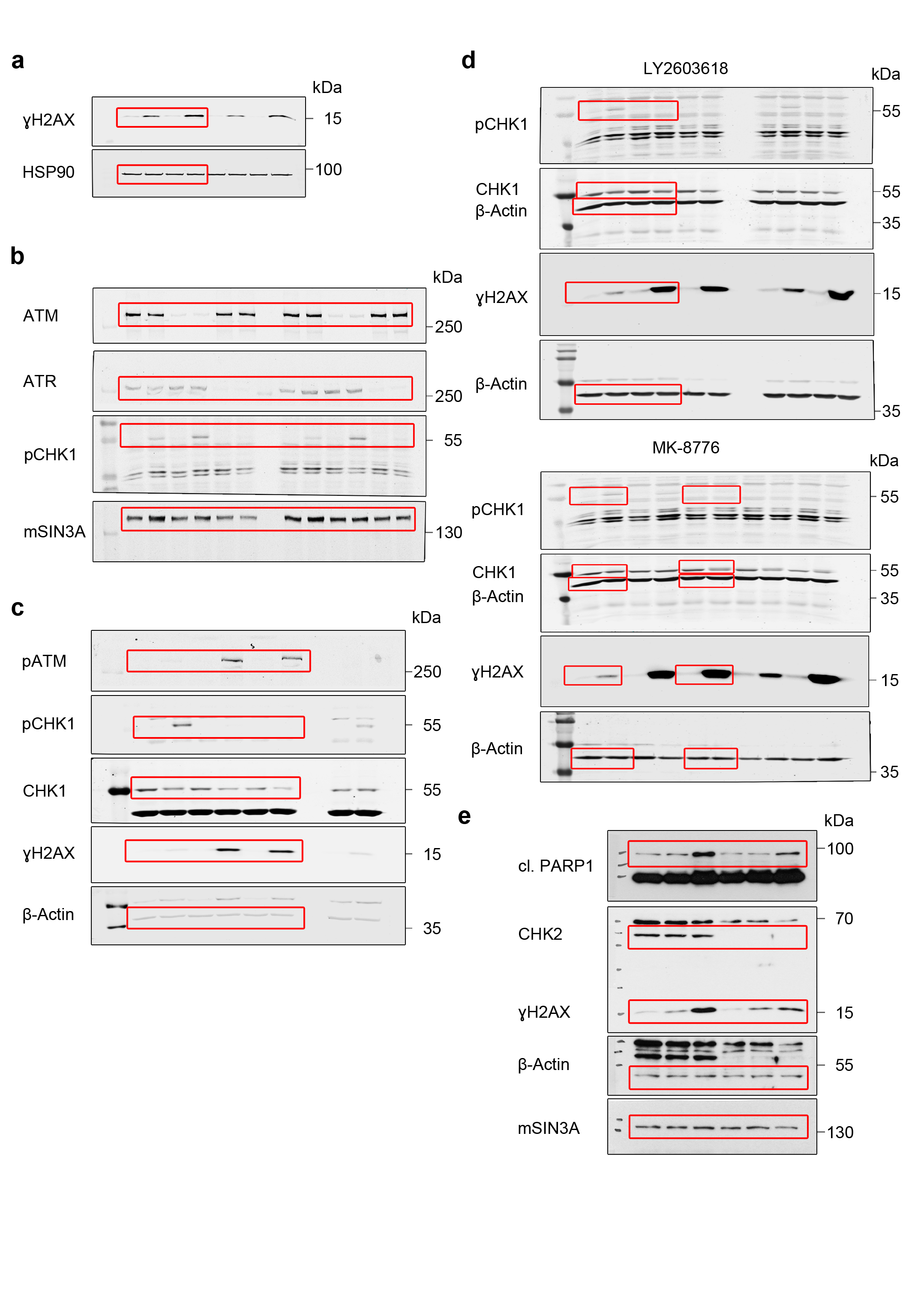


**Supplementary Figure 9. Uncropped Western blot images for data shown in Figures (a) 4b, (b) 4d, (c) 4f, (d) 4h, and (e) 4i.** Red boxes mark the parts of the Western blot images that are shown in the indicated figures.


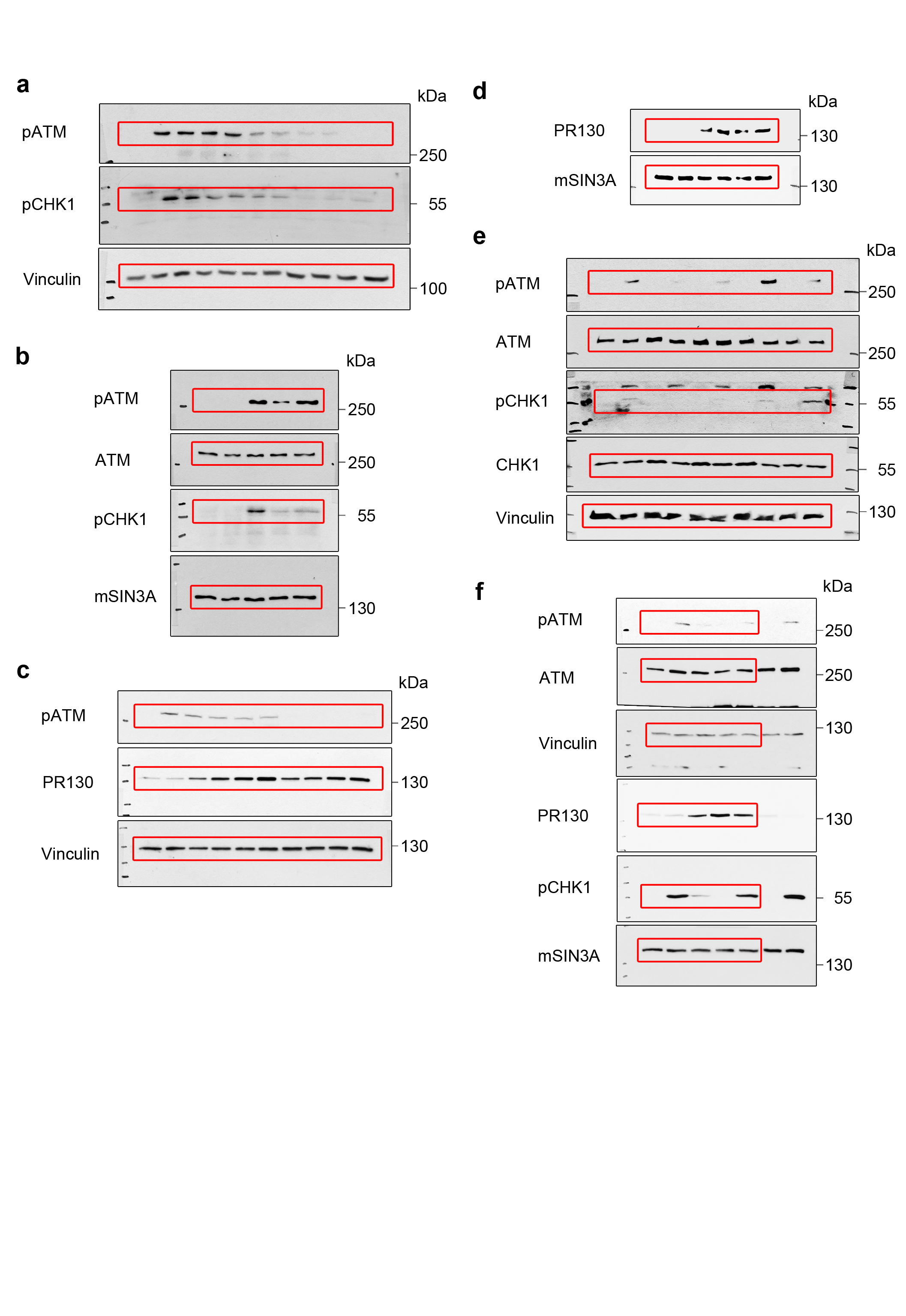


**Supplementary Figure 10. Uncropped Western blot images for data shown in Figures (a) 5a, (b) 5b, (c) 5d, (d) 5e, (e) 5g, and (f) 5h.** Red boxes mark the parts of the Western blot images that are shown in the indicated figures.


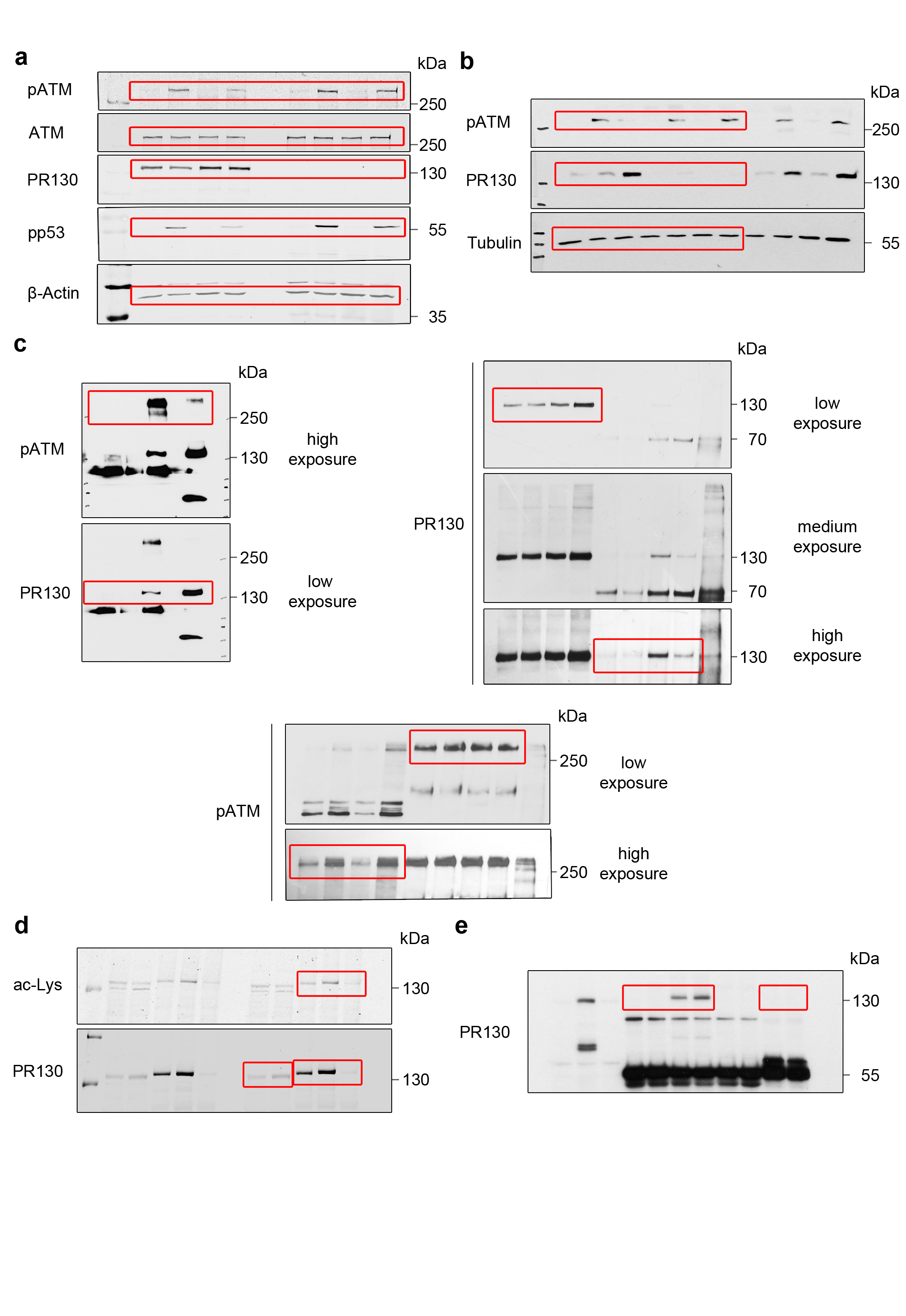


**Supplementary Figure 11. Uncropped Western blot images for data shown in Figures (a) 6b, (b) 6c, (c) 6d, (d) 6e, and (e) 6f.** Red boxes mark the parts of the Western blot images that are shown in the indicated figures.


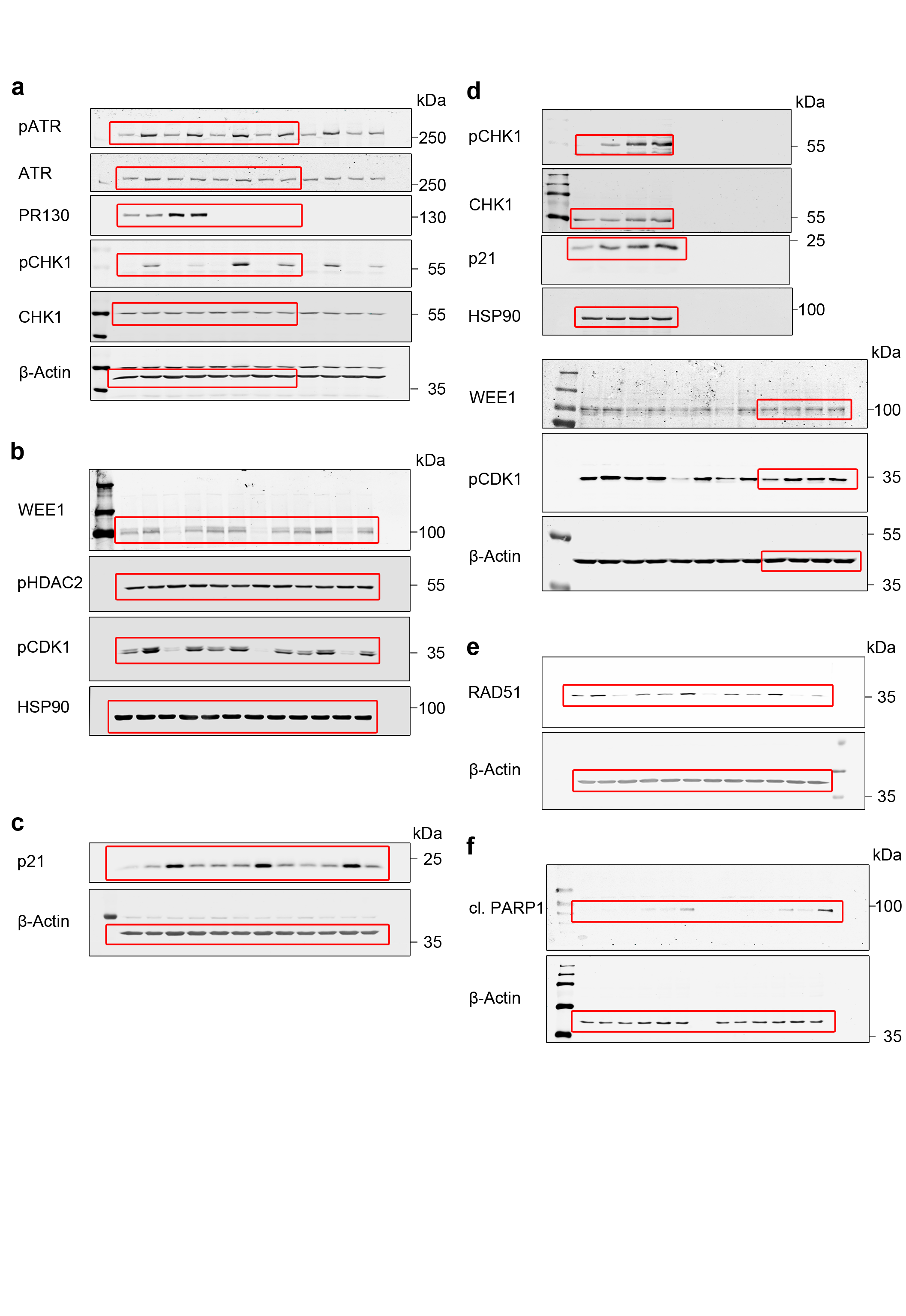


**Supplementary Figure 12. Uncropped Western blot images for data shown in Figures (a) 7a, (b) 7d, (c) 7e, (d) 7g, (e) 8d, and (f) 8e.** Red boxes mark the parts of the Western blot images that are shown in the indicated figures.
